# Techno-economic assessment of co-production of edible bioplastic and food supplements from Spirulina

**DOI:** 10.1038/s41598-023-37156-3

**Published:** 2023-06-22

**Authors:** Bushra Chalermthai, Pongtorn Charoensuppanimit, Kasidit Nootong, Bradley D. Olsen, Suttichai Assabumrungrat

**Affiliations:** 1 Bio-Circular-Green-economy Technology and Engineering Center, BCGeTEC, Department of Chemical Engineering, Faculty of Engineering, Chulalongkorn University, Bangkok, 10330 Thailand; 2 Control and Systems Engineering Research Laboratory, Department of Chemical Engineering, Faculty of Engineering, Chulalongkorn University, Bangkok, 10330 Thailand; 3 Department of Chemical Engineering, Massachusetts Institute of Technology, 77 Massachusetts Avenue, Cambridge, MA 02139 USA; 4 Center of Excellence in Catalysis and Catalytic Reaction Engineering, Department of Chemical Engineering, Faculty of Engineering, Chulalongkorn University, Bangkok, 10330 Thailand

**Keywords:** Chemistry, Engineering

## Abstract

Large amount of plastic wastes harming the environment have raised concerns worldwide on finding alternatives to non-biodegradable plastics. Microalgae has been found as a potential source for bioplastic production, besides its more common application in the pharmaceutical and nutraceutical industry. In this study, the objective was to techno-economically evaluate the large-scale co-production of Spirulina powder as food supplements and edible bioplastic for food packaging. The scale of production was large enough to satisfy 1% of local (Thailand) plastic demand (i.e., approx. 1200 MT y^−1^), and 1% of the global Spirulina demand (approx. 1000 MT y^−1^) as food supplements. Results showed that the co-production of the Spirulina powder and bioplastic revealed an attractive venture with a payback time (PBT) as low as 2.6 y and ROI as high as 38.5%. This was because the revenues generated were as high as US$ 55.6 million y^−1^, despite high capital (US$ 55.7 million) and operating (US$ 34.9 million y^−1^) costs. Sensitivity analysis showed differences in the profitability based on variations of major parameters in the study, where the split ratio of biomass used for food supplement versus bioplastic production and the bioplastic’s selling price were found to be the most sensitive.

## Introduction

In recent years, topics related to sustainability necessitate application of environmentally friendly technologies to satisfy the food-water-energy nexus. This includes development and exploitation of renewable sources for applications in energy, food, and bio-based products. However, utilizing food products which are first-generation feedstocks to produce energy have raised concerns regarding food versus fuel debate. In this framework, microalgae, which is a third-generation feedstock, that does not compete with food crops to produce food or energy, have received increasing interest as valuable feedstock for biofuels production, including biodiesel, biogas, bioethanol, and biohydrogen^1–4^. In addition, microalgae also have the potential to be used for carbon sequestration and production of other construction materials^5–9^. However, as far as economy is concerned, utilizing microalgae for single purpose or product has not yet been found to be economically feasible^10,11^. Hence, microalgal biomass set a huge potential for multiple valorization options, as they possess various valuable chemical compounds that can be converted or processed into other high-value products, namely pharmaceuticals and nutraceuticals, and bioplastics.

Among other microalgae, Spirulina (*Arthrospira* spp.) is distinctively important due to its high nutritional value that can be commercialized. It contains large amount of protein, carotenoid, and phycocyanin, and accounts for more than 30% of the biomass produced worldwide^12^. Spirulina is a cyanobacteria (blue-green microalgae), that is phototrophic, filamentous, and multicellular, and has characteristic photosynthetic capability^13^. The three most important species that are the most intensively investigated, due to their edibility and high nutritional value include: *Arthrospira platensis*, *Arthrospira maxima*, and *Arthrospira fusiformis*^14^. Spirulina contains several macro- and micro-nutrients, in addition to essential amino acids, lipids, and antioxidants, and is therefore known as a superfood^15^. Moreover, Spirulina is among the richest sources of proteins, with a protein content of about 60–70% dry weight and is also notable for its contents of essential fatty acids (g-linolenic acid), vitamins, and minerals^14^.

Besides its common application as food supplements, Spirulina also has the potential to be used for production of other high value products, including bioplastics. Several studies have assessed the use of Spirulina for potential of bioplastic production^16–19^, but techno-economic assessments (TEAs) have not been carried out to assess its feasibility for large-scale production. In the past, using agricultural products to make bioplastics competed with the food industry since the bulk of bioplastics was created by fermentation of agricultural products such as maize, wheat, potatoes, rice, and soy^20^. Hence, it would be advantageous if the microalgal industry can also co-produce bioplastics, in addition to its food supplements, since there is increasing demand of plastics for packaging purposes. In a single country, for example, within Thailand itself, the amount of plastic wastes surpassed 6300 MT per day in 2020, compared to 5500 MT per day in the earlier year (a rise of 15%), due to the COVID-19 pandemic which led to more food packaging plastic wastes from delivery and take-away services^21^. The total amount of plastic products sold in 2019 in Thailand was 650,000 MT, 19% (i.e., 123,500 MT) of which was attributed to plastic films and sheets^21^. Due to such high demands, reducing the amount of plastic wraps and films waste may be difficult if alternatives to greener, more sustainable choices are not made available to consumers. Hence, Spirulina-based bioplastic is a great alternative that can not only help solve a country’s food security, but also beneficial to the country’s economy, environment, and society.

Bioplastics can be produced from Spirulina in different ways: combining the whole biomass with glycerol, or extraction of protein and polysaccharides to produce protein-based and starch-based plastics, or extraction of PHAs (Polyhydroxyalkanoates) which are biopolymers found in the microalgal cells. In this study, the bioplastic production was simulated based on plasticization with glycerol, i.e. by combining dried microalgal biomass powder with glycerol to form bioplastic paste, and then extruded to produce plastic sheets. The basic mechanism for this conversion is provided in Supplementary Fig. S1. Previous studies have made use of Spirulina to produce bioplastics by utilizing glycerol as plasticizer, which are summarized in Table 1.

**Table 1:** Literature of Spirulina-based bioplastic plasticized with glycerol.

Microalgae strains	Plasticizers, compatibilizers and other additives	Operating conditions	Bioplastic type and characteristics	References
Spirulina and Chlorella with 46%-63% and 51%-58% protein content, respectively	Glycerol and polyethylene	4:1 biomass to glycerol ratio, 65%polyethylene/35% bio-blend; Thermochemical polymerization (injection molding)	Protein bioplastics with stress resistance of 3–5.7 MPa, strain resistance of 1.4–3.4%	^17^
			Chlorella showed more plasticity but Spirulina showed better blend performance	
*Arthrospira platensis*	Glycerol and maleic anhydride	Amount of maleic anhydride was varied: 0% wt, 2% wt, 4% wt, and 6% wt were used; Thermochemical molding	Protein bioplastics. Maleic acid amount of 6% wt gave bioplastic tensile strength of 28.26 kgf/cm^2^ (2.77 MPa) and elongation of 59.17%	^16^
Freeze dried *Arthrospira platensis* as filler	Glycerol, wheat gluten, octanoic acid and 1,4-butanediol	Microalgae biomass was added in: 10, 20, and 30 parts per hundred; Mechanical mixing and hot press molding	Biomass increased the tensile modulus from 36.5 MPa to 273.1 MPa, tensile strength from 3.3 MPa to 4.9 MPa, and bioplastic surface sensitivity against water	^18^
A consortium of 50% *Scenedesmus obliquus*, 30% *Desmodesmus communis*, and the rest as cyanobacteria and *Arthrospira platensis*. Protein content of the consortium was about 48%	Glycerol	Three contents of Spirulina biomass (50, 55, 60 wt%) were used. Consortium microalgae was used at two compositional levels (50 & 68.3 wt%); Thermochemical polymerization (injection molding)	Protein bioplastics with glass transition temperature around 60 °C	^19^

In recent years, edible packaging made of seaweed or macroalgae has also been picked up. Few notable brands selling seaweed-based edible packaging include Notpla and Evoware^22,23^. Since these are commercialized brands, no technical information has been provided on the type of macroalgae used or how the plasticization was done. Nevertheless, information on its pricing has been used as reference for techno-economic assessment (TEA) in this study.

To the best of our knowledge, no studies have been done on assessing the technical and economic aspects of large-scale microalgal biorefinery for the co-production of food supplements and edible bioplastic from Spirulina, hence it is the novelty of this research work to perform such assessment. The objective of this research, i.e., valorization of microalgae to produce high-value food supplements and edible packaging or bioplastic, is also in line with the UN Sustainable Development Goal no.12, which is to ensure sustainable consumption and production patterns of natural resources^24^. Although most of the information is based on geographical aspects of Thailand, the findings in this study are adaptable to other countries and regions that have similar climatic conditions which are suitable for the growth of the Spirulina. Sensitivity analysis is also conducted on parameters (such as scale of the project and selling price of the products) to analyze the changes in the profitability of the project in case of variations to the main assumptions used in the base case scenario.

## Methodology

### Data collection

In this study, the process design and planning were carried out based on data collected from different sources. For instance, experimental data was based off existing literature and used for scale-up calculations, current market prices of the products were referred from up-to-date websites related to market and economy, equipment sizing used in the simulation was based on realistic information from suppliers and manufacturers of machines and equipment, and information on local microalgal cultivation and agriculture from discussion with experts in the field. Wherever appropriate, all of the information was cited throughout the article.

### Selection of plant location

The simulation in this study was carried out to study a newly developed Spirulina farm so selection of the plant location is important. The location for the Spirulina biorefinery was assumed to be at higher elevation in a mountainous area of Chiangmai, a northern province of Thailand. The reasons for selecting this location include:

- Tropical climate is favorable for the cultivation of microalgae (average temperature of the region ranges between 22 and 36 °C)^25^.
- Direct sunlight is available all year-round (solar irradiance of approximately 11–13 h daily)^25^.
- At higher elevation, the farm can be less exposed to smog and pollution from the city center.
- An existing large-scale (40,000 m^2^) Spirulina farm called “Boonsom Farm” ^26^ is also located in this region so realistic assumptions can be made with regard to its successful mass cultivation.

### Software

SuperPro Designer (Academic Site Edition) version 12 build 3 (Intelligen Inc., Scotch Plains, NJ, USA) was used for the simulation of the Spirulina biorefinery. The program is equipped with mathematical models for the operation of various procedures that perform material and energy balances and equipment sizing calculations. It is a comprehensive process simulator that facilitates modeling, evaluation, and optimization of a wide variety of chemical, pharmaceutical, food, and related processes.

### Simulation process and associated assumptions

In this study, Spirulina biorefinery for co-production of two products: Spirulina powder to be sold as food supplements, and Spirulina-bioplastic as edible, biodegradable plastic wraps, was modelled. The model plant was operated in batch/semi-continuous mode, with an annual operating time of 330 days/year, accounting for plant maintenance and cleaning of 35 days/year. The project lifetime was set at 10 years.

The model was simulated for producing approximately 1000 MT y^−1^ of Spirulina powder and 1200 MT y^−1^ of edible-grade bioplastic, which would satisfy approximately 1% of the global and local demand, respectively^27,28^. Sensitivity analysis was also performed to address the variation in the amount of production, the ratio of each product produced, and their selling prices.

**Figure 1:**
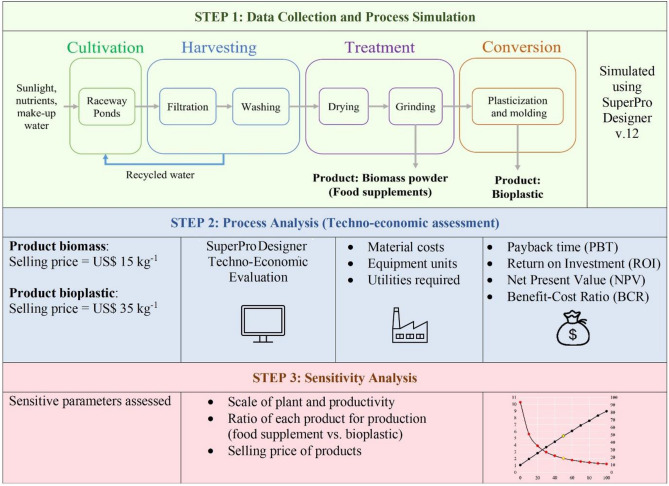
Overview of the systematic framework applied in the design of the Spirulina biorefinery for the co-production of biomass powder (food supplements) and edible bioplastic.

**Figure 2:**
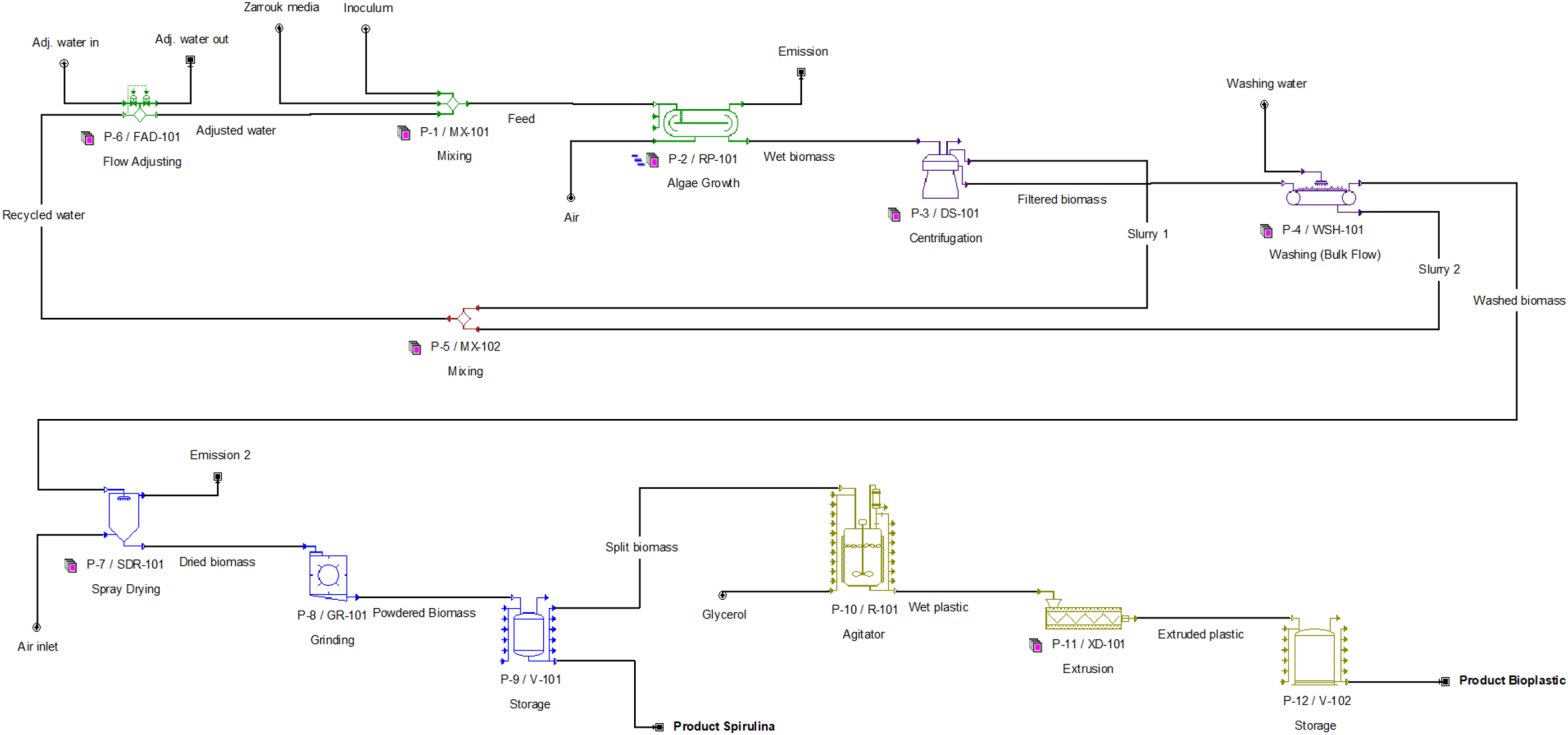
Process flow diagram of the Spirulina biorefinery using SuperPro Designer software.

The overview of the steps carried out in this study is shown in Fig. 1. The process flow diagram from SuperPro Designer is as shown in Fig. 2 and the modelling process associated with the unit procedures and equipment used for each process from cultivation until manufacture of final products are described in detail in Table 2.

**Table 2:** Description of processes and unit procedures used in the simulation.

Section	Equipment	No. of Units	Unit Operation	Description
1. Cultivation	P-1/MX-101	4	Mixing	• Temperature: 30 °C • Inlet of 6000 MT of water • Inlet of 60 MT of nutrients (1% of water) • Inlet of 60 kg of initial *Spirulina* inoculum (conc. 0.01 g L^−1^) • Mix all inlet streams to be used as feed for raceway ponds
	P-2/RP-101	16	Algae Growth	• Staggered raceway ponds (2 units of 8 ponds = 16 ponds) • Process time: 8 d • Pond size: 500 × 25 × 0.3 m^3^ • Reaction: 6000 Water + 60 ZM 60 Biomass + 6000 Slurry • Areal productivity (fresh biomass): 78.4 MT ha^−1^ y^−1^
2. Harvesting	P-3/DS-101	11	Disc-Stack Centrifuge	• Solids removal at 100 m^3^ h^−1^ • Centrifugation time: 6 h • Limiting solid particle size: 8 µm (diameter of Spirulina) • Particulate concentration: 100 g L^−1^
	P-4/WSH-101	43	Washing	• Set wash time the same as filtration time • Washer capacity: 2500 kg h^−1^
3. Recycling	P-5/MX-102	4	Mixing	• Combining two slurry streams for recycling
	P-6/FAD-101	3	Flow adjusting	• Adjust water amount for feed
4. Treatment	P-7/SDR-101	2	Drying	• Temperature: 70 °C • Time: 12 h • Final product LOD: 7% (moisture content of *Spirulina*) • Drying capacity: 2000 kg h^−1^
	P-8/GR-101	1	Grinding	• Time: 12 h • Max. grinding capacity 1000 kg h^−1^
	P-9/V-101	1	Storage	• Cool to final temperature: 25 °C • Storage time: 12 h • Split (50:50) for 2 purposes ○ Sell product as powdered biomass (food supplement) ○ Further processing for bioplastic production • Outlet product stream: Approx. 3.13 MT per batch of *Spirulina*, to satisfy demand of approx. 1000 MT y^−1^
5. Bioplastic production	P-10/R-101	1	Agitator	• Inlet glycerol at 1:4 of inlet biomass (w/w) • Reaction: 80 Biomass + 20 Glycerol 100 Bioplastic
	P-11/XD-101	3	Extrusion	• Temperature: 150 °C • Extrusion time: 4 h • Extrusion capacity: 600 kg h^−1^
	P-12/V-102	1	Storage	• Store for 1 h and released for sale

### Cultivation

Cultivation stage of Spirulina included several factors for consideration; how and where it was grown, and what would be needed for its growth. These factors are crucial not only for the highest productivity of the biomass aimed to be obtained, but also to fulfil the economic feasibility process which is the objective of this research. The productivity of Spirulina growth is dependent on environmental factors, including geographical location and temperature. In this study, Spirulina was simulated to grow in raceway ponds, since this was more economically feasible than closed photobioreactor types for large scale plant^29–31^. Open pond systems are also easy to clean and maintain, has direct exposure to sunlight, and has low accumulation of dissolved oxygen^12^. The cultivation period of the microalgae was specified by the process time of the raceway pond, which was set as 8 days, since maximum growth occur during day 8–9 after media addition^32^. Water supplemented with 10% (v/v) of Zarrouk medium was used to cultivate Spirulina^33^. An inoculum of the biomass at 0.1% of the nutrients was also added to the feed stream. The reaction in the raceway pond was specified as per Eq. (1) (weight basis) below:

1 $$6000\,\,{\text{Water}}\, + \,60\,{\text{Z}}\, \to 60\,{\text{Biomass}}\, + \,6000\,{\text{Slurry}}$$

where water is the tap water (US$ 0.85 m^−3^) used for food-grade production of biomass, ZM is Zarrouk medium (US$ 0.08 L^−1^), biomass is the product Spirulina, and slurry is the remaining water and nutrients mixture that are not used up and can be recycled for further cultivation. Hence, Eq. (1) referred to utilizing 6000 MT of water, supplied with 60 MT of ZM nutrients (1%), to produce 60 MT of biomass (1 g L^−1^), and the rest as recyclable slurry. These numbers were selected in order to achieve the target amount of products that satisfy 1% of the global Spirulina food supplements and 1% of local (Thailand) bioplastic demands. The amount of production was also varied in sensitivity analysis by varying the amount of the inlet streams. It was expected that recycling helped in reducing water footprint and energy consumption of the Spirulina production^34,35^. The cost of the ZM was based on its approximate price^12^ and tap water from water tariff rate of Thailand^36^. A summary of the prices of all the raw materials and chemical composition of ZM could also be found in Supplementary Tables S1 and S2, respectively.

Since the program model was set as batch mode and each batch took 11 days, running different batches consecutively one after the other would result in insufficient production of biomass. Hence, the raceway pond (P-2/RP-101) was set to be in staggered mode, with 15 extra equipment (i.e. the model has 16 ponds), in order to reduce the recipe cycle time, thereby led to an increase in the number of annual batches. The equipment was set in design mode, so the program automatically calculated the total amount of units required for the simulation. Given the pond configuration as described in Table 2, the amount of feedstock used in the base case scenario here was 6000 MT and the process time was 8 days, so 16 ponds (for simplicity of calculation) were used for the simulation process. The pond measured about 500 m in length by 25 m in width, so 16 ponds would require land area of about 200,000 m^2^ (or 20 ha). By including other machinery and equipment, the total land area for the plant would be approximately 22 ha. Given the productivity of biomass as specified in Eq. (1), the areal productivity of the fresh biomass was calculated to be 78.4 MT ha^−1^ y^−1^, at 1 g L^−1^ cell concentration. This was a reasonable productivity since areal productivity of microalgae grown in raceway ponds usually ranged between 33.1 and 82.7 MT ha^−1^ y^−1^ ^37,38^.

### Harvesting

After cultivation, the biomass was harvested and transformed into products by additional processing steps. In the harvesting stage, the objective was to reduce the amount of water to a concentration of between 10 and 25% solids by weight^39^. When the pH remained around 10 for about 24 h and the biomass turned dark green and looked thick in the pond, then it could be harvested. Filtration and washing of the biomass contributed to the harvesting stage of the Spirulina biorefinery.

Microalgal biomass might be filtered by different methods, depending on the physical and chemical characteristics of the microalgal species. Spirulina has individual cell diameter that measures 8 µm^40^, so disc stack centrifuge (P-3/DS-101) was used, since it was suitable for the separation of microalgae with particle sizes between 3 and 30 µm^41,42^. Moreover, disc stack centrifuges are the most common industrial centrifuge and are widely used in commercial plants for high value algal products and in algal biofuel pilot plants^42^. Centrifugation with capacity of 100 m^3^ h^−1^^43^ was used to obtain Spirulina paste with a concentration of 100 g L^−1^. Next, the filtered biomass was then washed to remove salt and impurities with clean water. The wash capacity was 2500 kg h^−1^^44^, which resulted in 43 units of the equipment required. The remaining washed stream and slurry from the centrifuge were then recycled for use as feed water, which were adjusted in P-6/FAD-101 to maintain appropriate amount for the next cultivation.

### Treatment

Once the Spirulina biomass was filtered and washed, they were then treated in order to improve the shelf life of the product. Wet biomass could also be sold directly, but more processing steps associated with its storage at low temperature and transportation in refrigerated containers would have to be taken into account. In this study, the biomass was treated and sold as dried powder, so there was no need for wet biomass storage and transportation steps. The amount of wet biomass after drying lost its moisture and approximately 50% of its weight was assumed to be retained as dried biomass with moisture content of about 7–8%. Spray dryer with drying capacity of 2000 kg h^−1^^45^ was used as this was suitable for large-scale biorefinery, due to its higher drying capacity and cost-effectiveness, compared to drum dryer or freeze-dryer^12^.

Once dried, the Spirulina biomass was then ground to be sold as Spirulina powder, and to be further processed for the production of bioplastic. The ground biomass was first cooled to 20 °C (since the stream was at 70 °C post-drying) in a storage silo before it was split for two products to be sold. The split for the base case scenario was 50:50 for biomass product versus for bioplastic production, as this yielded approximately 1% of the total biomass demand in the world (i.e. approx. 1000 MT y^−1^), and 1% of the total bioplastic wraps demand in Thailand (i.e. approx. 1200 MT y^−1^)^27,28^ which was a reasonable amount and size of the plant. Sensitivity analysis was performed on this parameter. Spirulina selling price was set at US$ 15 kg^−1^, based on the average bulk price sold online (i.e., US$ 10–20 kg^−1^)^46,47^, which was cheaper compared to retail commercial brands sold in Thailand (Market Price US$ 90–95 kg^−1^ ^48,49^) and in the US, at US$ 50–90 kg^−1^^50–52^. Sensitivity analysis was also performed on the selling price to assess its economic feasibility with varying pricing conditions.

### Bioplastic production

Microalgal bioplastic could be produced via different pathways. The one chosen for this study was based on the production of food-grade, clean plastic wraps, that are edible, biodegradable, and the least energy-intensive. The bioplastic production from Spirulina was based on the study by Zeller et al. ^17^, where glycerol (US$ 0.5 kg^−1^), which was used as plasticizer, was blended with Spirulina at high temperature, i.e. 150 °C, for 20 min. The reaction (weight basis) specified in the agitator P-10/R-101 was given by Eq. (2), where 4:1 (w/w) amount of the Spirulina biomass:glycerol was used for the production of bioplastic blend.

2 $${8}0{\text{ Biomass }} + { 2}0{\text{ Glycerol}} \to {1}00{\text{ Bioplastic}}$$

Glycerol could also be obtained from biodiesel industry as it is a major by-product in the biodiesel production via transesterification^53^. In the study by Zeller et al.^17^, compression molding was performed on the bioplastics to obtain dog-bone sheets for mechanical characterization^17^. The tensile property of the produced bioplastic was comparable to other edible bioplastics produced from biomass such as whey protein, soy protein, cassava starch, etc.^17,54–56^.

In this study, extrusion method was chosen to produce microalgal bioplastic. Extrusion is a classical thermoplastic-processing tool for mixing and/or plasticizing polymers and obtaining homogeneous blends^57^. The advantages of extrusion process compared to injection molding include its flexibility of design where different shapes (pipes, tubes, sheets, etc.) of plastic materials can be obtained, as well as its feasibility for high volume manufacturing ^58^. 1:4 w/w amount of glycerol: Spirulina was first blended for 3 h in the agitator P-13/R-101 (Fig. 2), that has a working volume of up to approx. 8500 L. The homogeneously blended material was then extruded at 100 rpm, at 150 °C^17,59^ for 4 h (approx. 400 kg h^−1^). The specific power consumption was set at 0.1 kW kg^−1^ h^−1^, which is typical for extrusion of polymeric material (between 0.08 kW and 0.16 kW kg^−1^ h^−1^) ^60^. The final Spirulina-bioplastic was then cooled down in a storage unit before being sold at a unit selling price of US$ 35 kg^−1^. This selling price is low, compared to that of edible grade, algal (seaweed)-based bioplastic produced by Evoware and Notpla^22,23,61^, which ranges from US$ 30–250 kg^−1^, assuming that bioplastic sheets weigh 50–100 g m^-2^. Sensitivity analysis was also performed on the selling price to assess the economic feasibility in case the bioplastic is sold at different price ranges due to varying quality and purposes.

Since the amount of the bioplastic produced was slightly higher than that of the powder Spirulina, the product bioplastic was set as the main product of the simulation of the process, where profitability analysis and calculations were performed on. Sensitivity analysis was also performed on the selling prices of both the products to evaluate the economic feasibility of the plant process.

### Economic evaluation

The total capital cost or expense (CAPEX) and annual operating expense (OPEX) of the plant were calculated based on different parameters. The CAPEX or the total capital investment refers to the fixed costs that are associated with a process. This was calculated as the sum of the following cost items over all sections of a process: direct fixed capital (DFC), working capital, start-up and validation cost, and up-front R&D cost. Components of the DFC include contractor’s fee and total plant cost, which was based on the total equipment purchase cost (PC) in SuperPro Designer or from well-established values found in literature, which are: piping (31%), instrumentation (28%), electrical facilities (10%), buildings (22%), and construction (34%) (all from SuperPro Designer default values); and insulation (3%), yard improvement (10%), engineering/supervision (25%), and auxiliary facilities (40%) (all from^62^). The formulation for the calculation as well as the results obtained for CAPEX are provided in Supplementary Table S1. On the other hand, the OPEX or the Annual Operating Cost (AOC) of a project included costs that are related to the demand for a number of resources (i.e., raw materials, consumables, labor, heating/cooling utilities and power), as well as additional operational costs. More specifically, the OPEX was calculated as the sum of the following cost items: materials cost, consumables cost, labor-dependent cost, utilities (heating/cooling utilities and power) cost, waste treatment/disposal cost, facility-dependent cost, laboratory/QC/QA cost, transportation cost, miscellaneous costs, advertising/selling costs, running royalties, and failed product disposal cost. Other costs such as income taxes (20%), NPV interest rate (2%), inflation rate (4%), and land leasing (included under miscellaneous cost of OPEX) are included in the calculation based on Thailand data^63–66^.

The project lifetime was assumed to be 10 years, with 100% operating capacity in every year, and a product failure rate of 1%. The labor wage rate was calculated to be US$ 23 h^−1^ for all workers, based on a conservative basic rate of US$ 10 h^−1^, and the time estimation of work hours was 60% of the batch processes. The aqueous waste disposal and treatment cost was specified as US$ 0.11 m^−3^ of treated effluent, which is the average wastewater treatment cost in Thailand^67^. The land price was estimated based on Chiangmai land purchase price in Mae Rim area of THB 3.5 million per *Rai* (1 *Rai* = 6.25 ha)^68^, and therefore the land price for 22 ha was US$ 14.1 million, or leased at US$ 1.41 million y^−1^, for the project lifetime of 10 years.

### Profitability parameters

The tecno-economic assessment of the study was carried out and the profitability was measured and discussed to evaluate the viability of the investment. The four main parameters used to evaluate the profitability in this study were: payback time, return on investment, net present value, and benefit–cost ratio.

### Payback time (PBT)

The PBT is a measure of the time needed for the total capital investment to be exactly balanced by the cumulative net profits. The project would be more attractive at shorter PBT. It was calculated by dividing the total capital investment by the annual net profit (Eq. 3).

3 $$\text{PBT }\left({\text{y}}\right) \, = \text{ } \frac{{\text{CAPEX}} \, }{\text{Net profit}}$$

where

4 $${\text{Net}}~\,\,{\text{Profit~}}\,\,(\$ )\,~ = \,~{\text{Total}}\,\,{\text{Revenues}}~ - {\text{OPEX}} - ~{\text{Income~}}\,\,{\text{Taxes~}}\, + \,~{\text{Depreciation}}$$

where the total revenues refer to the sales of all of the products, OPEX to annual operating cost, income tax of 20%, and depreciation which was an income tax deduction that represents a fixed capital loss (which is mostly due to equipment wear out and obsolescence spread over a predefined depreciation period (i.e. 10 years)). More details on depreciation and its equation can be found in the Supplementary information section S1.

### Return on investment (ROI)

The ROI is another profitability measure used to evaluate the viability of an investment. If an investment does not have a positive ROI, or if there are other opportunities with a higher ROI, then the investment should not be undertaken. It is calculated by dividing the annual net profit by the total capital investment (Eq. 5).

5 $${\text{ROI }}\;(\% ) = \frac{{\text{Net profit}}}{{\text{CAPEX }}} \times 100$$

### Net present value (NPV)

The NPV is a profitability measure used to evaluate the viability of the investment. It represents the total value of future net cash flows during the lifetime of a project, discounted to reflect the time value of money at the beginning of a project (i.e., at time zero). If the NPV is negative, or if the investment has lower NPV compared to other scenarios, then the investment should not be undertaken. The NPV was calculated based on user’s specified interest rate as per Eq. (6).

6 $$NPV (US\$)= \sum_{k=1}^{n}\frac{NC{F}_{k}}{{\left(1+i\right)}^{k}}$$

where:

- i is the interest rate,
- NCF_k_ is the net cash flow in year k, and
- n is the project lifetime (in number of years).

### Benefit–cost ratio (BCR)

The BCR is the ratio of the present value of benefits to the present value of costs over the project lifetime (10 years). The BCR was calculated as the total cash benefit (revenues obtained) from the project, divided by the total cash cost of the project (Eq. 7). If BCR > 1, then the project is likely feasible, and vice versa.

7 $${\text{BCR}}\,({\text{US}}\$ )~ = ~\frac{{{\text{Total}}~\,\,{\text{revenues}}~\,\,{\text{by}}~\,\,{\text{the}}\,\,{\text{end}}~\,\,{\text{of}}\,\,~{\text{project}}}}{{{\text{Total~}}\,\,{\text{cost}}\,\,({\text{CAPEX}} + {\text{OPEX}})~\,\,~{\text{by}}\,\,{\text{~the}}\,\,~{\text{end}}\,\,~{\text{of}}\,\,{\text{the}}~\,\,{\text{project}}}}$$

## Results and discussion

### TEA results

The model was set to operate in batch mode in SuperPro Designer, with annual operating time of 330 days y^−1^. By splitting equal amount (split ratio = 50:50) of dried biomass from the equipment P-9/V-101, the program execution resulted in the production of 953 MT y^−1^ of Spirulina powder which could be sold as food supplements, and 1191 MT y^−1^ of bioplastic. The unit price of the products and their annual revenues are as summarized in Table 3.

**Table 3:** Summary of key parameters for the TEA performance.

Parameter	Value	Unit
General project assumptions		
Annual operating days	330	days y^−1^
Project lifetime	10	y
Biomass productivity and land required		
Areal productivity of Spirulina fresh biomass	78.4	MT ha^−1^ y^−1^
Dimension of 1 raceway pond	500 × 25 × 0.3	m^3^
No. of raceway ponds	16	
Land area for raceway ponds	20	ha
Total land area required (for ponds and other machinery)	22	ha
Products of the Spirulina biorefinery		
Split ratio for production of food supplement:bioplastic	50:50	
Amount of food supplement (Spirulina powder)	953	MT y^−1^
Amount of bioplastic	1191	MT y^−1^
Unit selling price of food supplement	15	US$ kg^−1^
Unit selling price of bioplastic	35	US$ kg^−1^
Revenues of the project		
Annual revenue of food supplement	14.29	million US$ y^−1^
Annual revenue of bioplastic	41.28	million US$ y^−1^
Annual revenue of both products	55.57	million US$ y^−1^
Total revenues (end of project lifetime)	555.7	million US$
CAPEX and OPEX		
Total Capital Investment	55.67	million US$
Annual Operating Cost	34.93	million US$ y^−1^
Total Operating Cost (end of project lifetime)	349.3	million US$
Profitability of the project		
Benefit–Cost Ratio (BCR)	1.37	
Return On Investment (ROI)	38.46	%
Payback Time (PBT)	2.6	y
NPV (at 2% interest rate)	125.84	million US$

**Figure 3:**
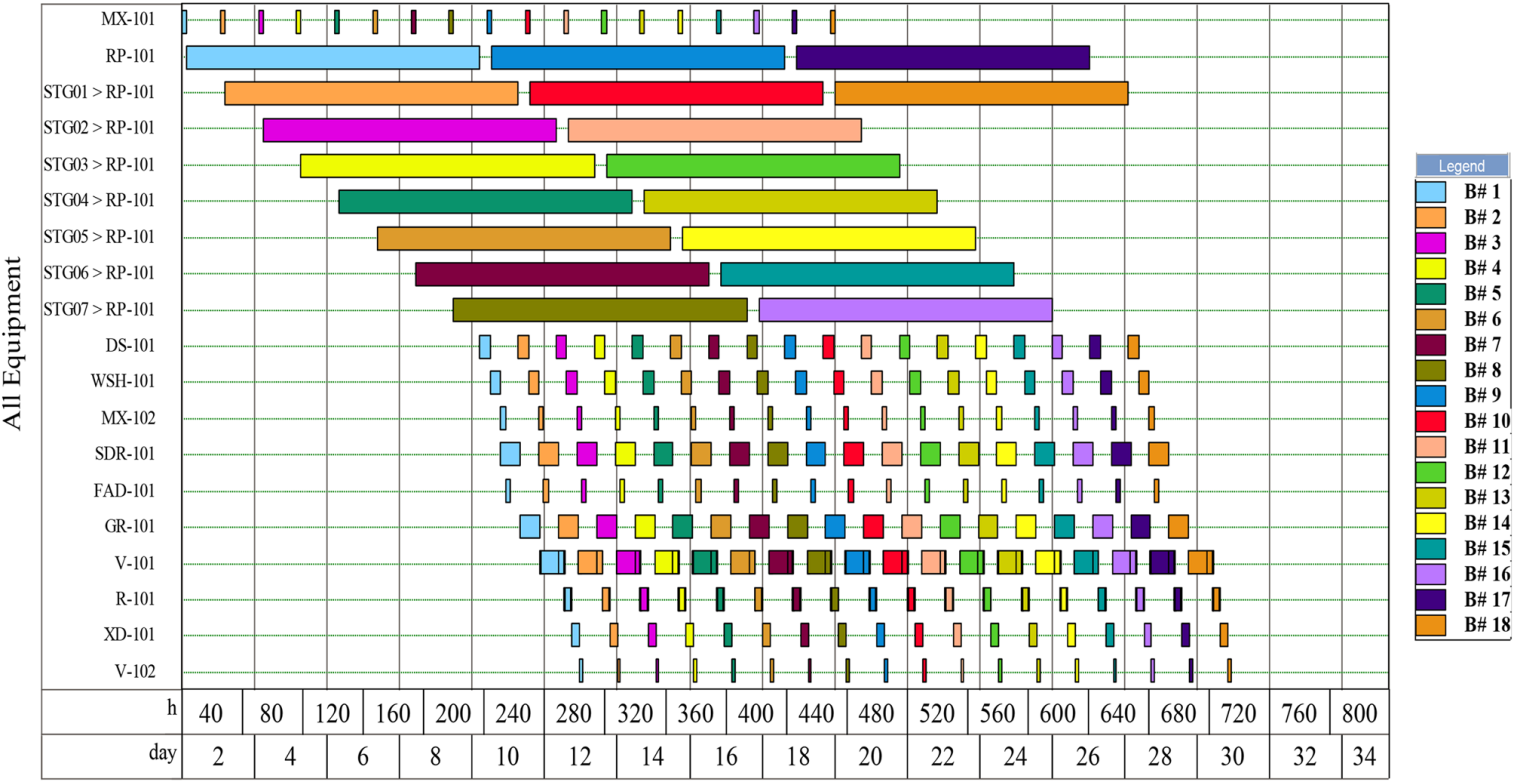
Equipment occupancy chart showing execution of the various processing steps as a function of time for 18 consecutive batches.

The total number of batches obtained was 304 per year, with an overall batch time of 264.93 h (i.e., 11.04 d). The recipe cycle time (time between consecutive batches) was 25.25 h, given that the time slack of 1 h was specified (for equipment resting). The minimum recipe cycle time (minimum time interval between consecutive batches) was 24.25 h and was determined by P-2 (in RP-101), which was the cycle time bottleneck, as it was the procedure with the longest duration. As shown in the equipment occupancy chart in Fig. 3, the RP-101 is in staggered mode, so that continuous processes of harvesting follow right after collection of biomass from the raceway ponds, without having to wait for the completion of 8 days process time in each pond before the next batch could commence.

In terms of economy, the CAPEX of the project amounted to a total of US$ 55.7 million, whereas the OPEX was totally US$ 34.9 million y^−1^. This is a large-scale investment, which is reasonable since considering production of 1% of the total Spirulina global demand, and 1% of the total plastic demand in Thailand. The contributions to the CAPEX and OPEX are provided in the supplementary information Tables S1 and S2. It was found that as much as 92% of the CAPEX (US$ 51.4 million) was due to the direct fixed capital cost (DFC). Meanwhile, the DFC was mainly contributed by the costs of equipment purchase (PC), plant construction, engineering, contingency, and equipment installation. Other contributions to the DFC was shown as pie chart in Supplementary Fig. S1. The construction cost and the PC contributed the most, at 19% each, of the DFC. This was similar to the study by Hossain et al. ^1^ where the PC also contributed to about 20.5% of the CAPEX for microalgae cultivation. The OPEX, on the other hand, was majorly contributed by the cost of raw materials, which amounted to US$ 17 million y^−1^ (49% of the OPEX), where Zarrouk media was the most costly of all (Supplementary Table S2).

**Figure 4:**
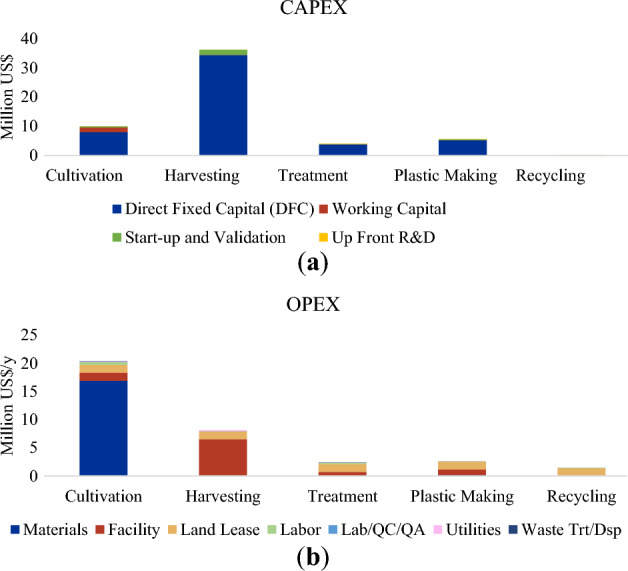
Results of the economic cost: (**a**) CAPEX and (**b**) OPEX of the Spirulina biorefinery.

Section-wise, it was found that the harvesting section contributed to majority (64%) of the CAPEX, followed by cultivation, and plastic-making sections (Fig. 4a). Meanwhile, the major contribution to the OPEX was the cultivation section, where material cost (Zarrouk medium) contributed the most (US$ 14.2 million y^−1^, i.e., 40% of the OPEX) (Fig. 4b). This was similar to the result found in the study by Grima et al. (2003) where the cost of culture medium accounted for as much as 35% of total cost of the algal biomass production ^69^. Cost of raw materials was also found to be significant in another TEA study on three microalgal strains by Lopes et al. (2023)^70^. It was found that the contribution was mainly due to the price of dipotassium phosphate used as nutritive media for the Spirulina cultivation^70^. In other studies, the harvesting and cultivation have also been found to be the most expensive stages. It was estimated by Barros et al. (2015) that the harvesting stage can represent between 20 and 30% of the production costs of microalgal biomass^39^. Meanwhile, in a study by Vonshak (1997), harvesting stage (including filtering, drying, and packing) costed about US$ 541,000, for a raceway-type reactor with land area of 50,000 m^2^, i.e. US$ 10.82 m^−2^ ^71^. This is similar compared to the result found in this study, where the working capital and start-up and validation costs for the harvesting stage amounted to US$ 1.74 million, for project area of approx. 22 ha (220,000 m^2^), which translated to areal cost of US$ 7.9 m^-2^.

The economic profitability is dependent on the revenues generated, in addition to the total costs incurred on the plant. The total revenues obtained from this project, as summarized in Table 3, was US$ 55.6 million y^−1^, whereby US$ 41.3 million y^−1^ (74% of total revenues) was generated from the sale of Spirulina-bioplastics (US$ 35 kg^−1^), and the remaining US$ 14.3 million y^−1^ (26% of the total revenues) from Spirulina powder sold as food supplements (US$ 15 kg^−1^). This resulted in a PBT of 2.6 years, with ROI of 38.5%, and NPV (at 2% interest) of US$ 125.8 million, which is very profitable. The BCR was also calculated for the end of the project lifetime (10 y) and was found to be 1.37. Since the BCR was greater than 1, the project is considered feasible and has great potential for investment. These results implied that given the possibility of high investment (over US$ 56 million as CAPEX and US$ 35 million y^−1^ as OPEX), the return and net gains was well-paid off. The biorefinery for the co-production of Spirulina powder for food supplements and edible bioplastic for food packaging, hence, would be attractive to investors who wish to work on such projects anywhere, given similar climatic and geographical conditions. The co-production of both food supplements and bioplastics was thus more profitable than that of the study by Bataller and Capareda (2022) where only single product (Spirulina powder) was obtained and photobioreactors were used rather than raceway ponds, in which the PBT was found to be 6.34 y^72^. However, several assumptions such as plant capacity and harvesting technique were also different, so taking these into account, some parameters and their assumptions may be adjusted accordingly to obtain even more positive and profitable outcomes. The key economic performance and profitability results are summarized in Table 3.

In terms of energy demand, it was found that the total energy required for the full-scale production (i.e. 953 MT y^−1^ of Spirulina powder, and 1191 MT y^−1^ of Spirulina-bioplastic) was 2.5 million kWh (Supplementary Table S6). This is approximately 1166 kWh MT^−1^ of the products (Spirulina powder and bioplastic combined), or 2100 kWh MT^−1^ of bioplastic product only, or 2623 kWh MT^−1^ of Spirulina powder only. The results were slightly lower compared to the values in literature, where the energy consumption for Spirulina production was found to be in the range of 3200–4260 kWh MT^−1^^73^. Most of the energy consumption was attributed to the harvesting stage (64.3%), followed by cultivation stage (19%), treatment (10%), and plastic making (7%) (Supplementary Table S7). The harvesting stage contributed to the most energy demand due to the use of the disc-stack centrifuge, at 1.6 million kWh y^−1^. This result was not unexpected since centrifuge machines are energy-intensive, especially when used for large-scale production ^42^.

In terms of environmental impact, 1166 kWh MT^−1^ of Spirulina products (powder and bioplastic combined) would correspond to 0.504 MT of CO_2_ eq. MT^−1^ y^−1^ ^74^. This is considered low, as it is equivalent to 215 L of gasoline consumed per year, or about 6.4% of home’s energy use in a year ^74^. In terms of carbon sequestration, 1166 kWh is also equivalent to carbon sequestered by 8.3 tree seedlings grown in 10 years ^74^. Spirulina production plant could be considered as carbon neutral, as found in the result of the study by Tzachor et al. (2022)^75^, where it was found that for every kg of wet biomass of Spirulina produced, about − 0.008 CO_2_ eq. is emitted (net zero). Therefore, Spirulina production plant can be considered sustainable as it is environmental-friendly with low carbon emissions.

### Sensitivity analysis

**Figure 5:**
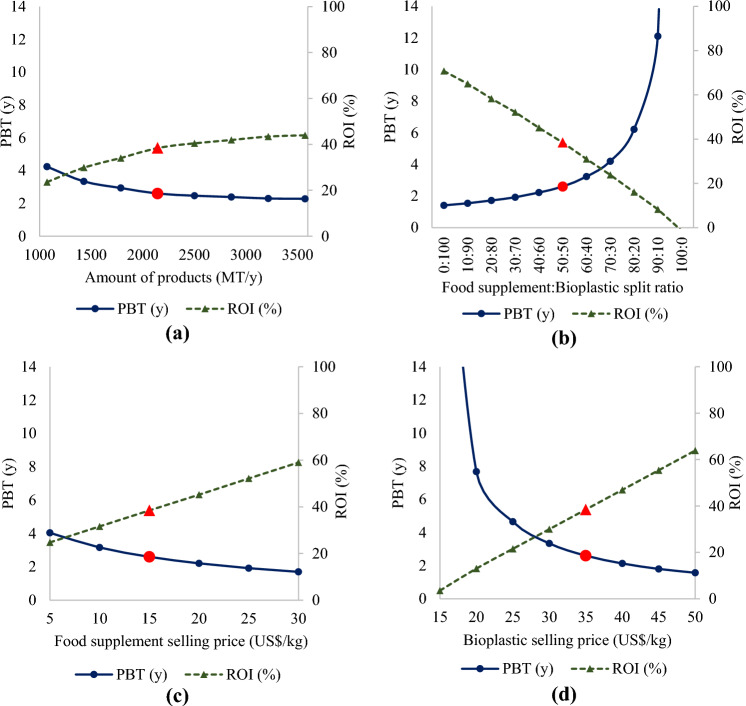
Sensitivity Analysis of the PBT (solid lines, round markers) and ROI (dashed lines, triangular markers) on four major variables: (**a**) Amount of products produced per year (indicating annual capacity of the plant); (**b**) Split ratio between Spirulina powder (food supplement) and bioplastic in equipment P-9/V-101; (**c**) Selling price of the product food supplement; (**d**) Selling price of product bioplastic. Red markers represent values for the base case scenario.

Sensitivity analysis was performed on four major variables that were expected to affect the profitability of the Spirulina biorefinery plant, while maintaining other variables constant at the base case scenario. The parameters were: (a) capacity or scale of the plant; (b) the percent split between the two products (Spirulina powder and Spirulina bioplastic); (c) selling price of Spirulina powder; and (d) selling price of Spirulina bioplastic. The results are as displayed in Fig. 5a–d.

The scale or capacity of the production plant could be determined by the amount of production of the Spirulina products, based on the amount used in the feed stream. The reason this parameter was chosen for sensitivity analysis was because variation in the amount of production could greatly affect the overall economic of the process, given other variables constant ^72^. However, as can be observed in Fig. 5a, the amount of production did not affect the profitability much as the PBTs were maintained below 5 years and ROI above 20% in all cases, when the production capacity varied from half the base case value, i.e. approx. 1000 MT, to almost double, i.e. approx.. 3500 MT annually (base case was 2144 MT y^−1^, marked with red markers). This showed that the profitability was quite constant despite the variation in amount of production.

The split ratio for the production of the two products was also analyzed for sensitivity on profitability measures. This was because the split ratio could affect the profitability since the unit selling prices and amount of production of both the products were not equal. Moreover, even at equal split ratio of 50:50, the mass of the products produced (MT y^−1^) were still unequal anyway, since the food supplement was purely Spirulina powder, whereas the bioplastic was the powder mixed with plasticizer (so the mass of bioplastic is 25% greater than that of pure Spirulina powder). The results showed that the split ratio from the equipment P-9 for the production of two products, i.e. Spirulina bioplastic and food supplements, affected the sensitivity of the plant quite significantly, as can be observed from the steepness of the slopes of the graphs of PBT and ROI in Fig. 5b. The PBT increased to over 6 y and ROI reduced to only about 16%, when the food supplement to bioplastic ratio was 80:20. If the ratio was increased to 90:10, then the project would be considered infeasible with over 12 y of PBT and ROI below 10%. Hence, this project would only be feasible with favorable PBT of below 3 y as long as the split ratio was at most 50:50 of food supplement:bioplastic. This could be attributed to the reason that food supplements could be sold at cheaper unit price compared to the bioplastics. Therefore, the bioplastic should be considered as the main product and should occupy at least half (or preferably, more percentage) of the total products amount in the co-production process. Compared to real-world scenario, Boonsom Farm which is also based in Chiangmai, Thailand ^26^, was able to commercialize fresh and dried biomass for food supplements, without co-production with any other high-value Spirulina-based products. This inconsistency with the real-world result could be attributed to the assumptions relevant to the scale of production (20 ha in our study to 4 ha in Boonsom farm), and the expensive land lease price in our study due to inflation, among other parameters. Therefore, if the scale of the plant could be reduced to 20% of the current study, using different sets of assumptions on ponds and machinery configurations, then co-production with bioplastic might not be necessary to achieve a positive and high ROI.

The last parameter picked for sensitivity analysis was the selling price of the products. This was because the market prices for both products vary greatly, based on different market locations globally ^22,46,47,61^. In our study, the results showed that the selling price of the Spirulina powder sold as food supplements could be as low as US$ 5 kg^−1^where the PBT could be maintained at below 5 y. The Spirulina powder could be sold at such low values, due to the co-production with bioplastic, which generated more revenues due to its higher unit selling price (US$ 35 kg^−1^). In a study by Bataller and Capareda (2022)^72^, the minimum selling price was also determined for different reactor’s capacity. It was found that the minimum selling price of Spirulina powder was US$ 6.12 kg^−1^ at 100 m^3^ photo-bioreactor capacity, where the PBT could be maintained at approx. 6.34 y. However, the scale of production in that study was also much lower, i.e. production of approx. 14.5 MT y^−1^ of Spirulina powder in 40–120 m^3^ airlift photobioreactors, and it was also expected that the price could be much lower for larger scale production. The selling price of bioplastic, on the other hand, was a more sensitive parameter, not only due to its greater production capacity (1191 MT of Spirulina bioplastic, compared to 953 MT of Spirulina powder, in the base case scenario), but also greater value for money (US$ 35 kg^−1^ of Spirulina bioplastic, compared to US$ 15 kg^−1^ of Spirulina powder, in the base case scenario). Its sensitivity can be observed in Fig. 5d where the slopes of the PBT and ROI graphs are much steeper, compared to another parameter such as the food supplement’s selling price in Fig. 5c. In order to maintain the PBT of the plant to below 5 y, then the minimum selling price of the edible bioplastic should not be below US$ 25 kg^−1^. The PBT increased significantly to 7.7 y and ROI 13%, when the selling price of the bioplastic was as low as US$ 20 kg^−1^, therefore would not be so feasible for the project of this scale.

### Challenges of the TEA study

Although the results obtained for this study showed to be techno-economically feasible due to high value of Spirulina products, further consideration must be taken if the project was to be implemented in reality. It is without doubt that TEA study generally involves up-scaling assumptions based on lab experiments and pilot-scale data, so positive outcome might not always be achievable for large scale commercial processes. Besides this general limitation, other challenges specific to this study also include:

- Open raceway ponds, unlike photobioreactors, tend to have lower productivity which could be due to CO_2_ losses and evaporation, contamination, temperature and seasonal fluctuations, among others^10,76,77^. Although in this study we assumed an achievable productivity of 78.4 MT ha^−1^ y^−1^ (areal productivity for raceway pond could be 33.1–82.7 MT ha^−1^ y^−1^^37,38^), this value might not be consistent throughout the project’s lifetime of 10 years. To address this effect, a high maintenance cost (as part of facility-dependent cost of the OPEX; Supplementary Table S2) was assumed, to cover for unforeseen circumstances that could occur during the project’s lifetime.
- In terms of bioplastic production within Thailand, bioplastic from other agricultural feedstock such as sugarcane bagasse and cassava might be more competitive due to its abundance of agricultural resources^78,79^. In that sense, Spirulina bioplastic might not be as competitive and investors might want to consider markets for export purposes instead. Nevertheless, Spirulina continues to have increasing demands with a compound annual growth rate (CAGR) of 9.4%^27^ so the future 10–20 years may see a shift towards its bioplastic production and commercialization.

Despite some of these challenges, the TEA has helped identify bottlenecks, major contributions to the costs, and appropriate scales for the market objective. Hence, the TEA is still a useful and crucial tool for the determination of the economic viability of the Spirulina biorefinery, especially since no studies have been carried out to assess its mass co-production of bioplastics and food supplements before.

## Conclusion

The co-production of Spirulina powder and Spirulina bioplastic was found to be economically feasible with a payback time as low as 2.6 y and ROI of 38.5%, when the production capacity aimed to satisfy 1% of the global and local (Thailand) demand of the Spirulina food supplement and plastic, respectively. Edible packaging made from Spirulina bioplastic will not only be beneficial to the overall economy, due to its greater market value, but also favorable for environmental sustainability. For more realistic approach, future studies may consider construction of the bioplastic plant adjacent to existing Spirulina farms, so that less land areas will be required and less assumptions can be made on the upstream processes (cultivation and harvesting) within the simulation. In addition, bioplastic production via different pathways, such as copolymerization process with proteins extracted from Spirulina, or plasticization with other plasticizers and solvents such as PVA (polyvinyl alcohol) may also be considered. Other lower cost media for the cultivation and less-energy intensive processes during harvesting may also be considered, to reduce the overall costs of the production and make the Spirulina biorefinery even more profitable.

## Supplementary Information

Supplementary Information.

## Data Availability

All data generated or analyzed during this study are included in this published article (and its Supplementary Information file).

## Abbreviations

AOC: Annual operating cost
BCR: Benefit-cost ratio
CAPEX: Capital expense/expenditure (s)
DFC: Direct fixed capital cost
LOD: Loss on drying
NPV: Net present value
OPEX: Operating expense/expenditure (s)
PBT: Payback time
PC: Purchase cost of equipment
ROI: Return on investment
TEA: Techno-economic assessment
ZM: Zarrouk medium
