# Supplementary material for: Techno-economic assessment of co-production of edible bioplastic and food supplements from Spirulina
Source: Sci Rep. 2023 Jun 22;13:10190. doi: 10.1038/s41598-023-37156-3 (PMC10287645; doi:10.1038/s41598-023-37156-3)
Supplement: Supplementary file 1 — Supplementary Information. [file 41598_2023_37156_MOESM1_ESM.pdf]

Supplementary/Supporting Information

For the Manuscript

Techno-economic assessment of co-production of edible bioplastic and food supplements from Spirulina

Authors: Bushra Chalermthai, Pongtorn Charoensuppanimit*, Kasidit Nootong, Bradley D. Olsen, Suttichai Assabumrungrat

*Corresponding author: pongtorn.ch@chula.ac.th

The Supplementary information below is divided into 3 main sections: supplementary figures, supplementary tables, and other supplementary information text.

Supplementary Figures

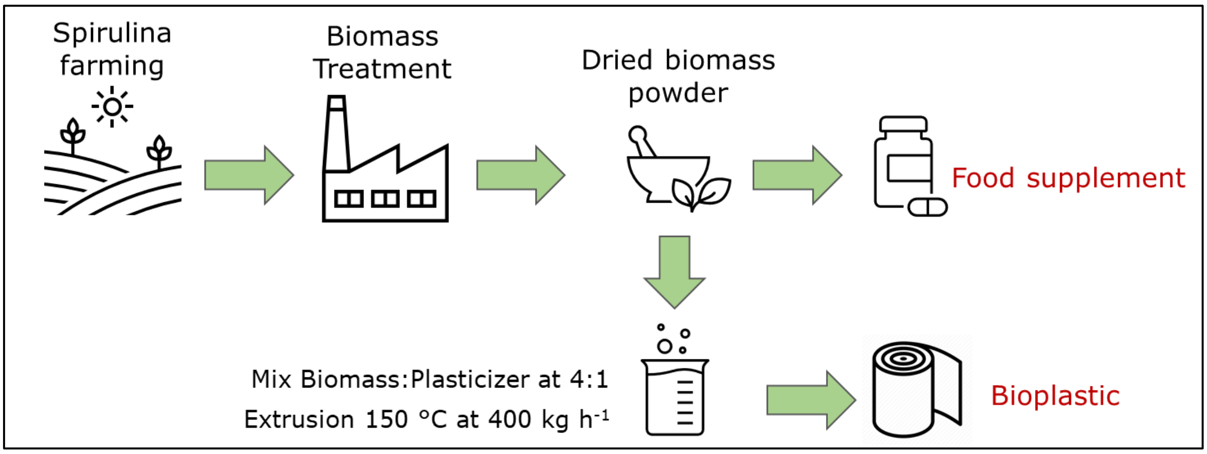

Figure S1. Overview of the Spirulina biorefinery with basic mechanism for the conversion of dried biomass to bioplastic used in this study.

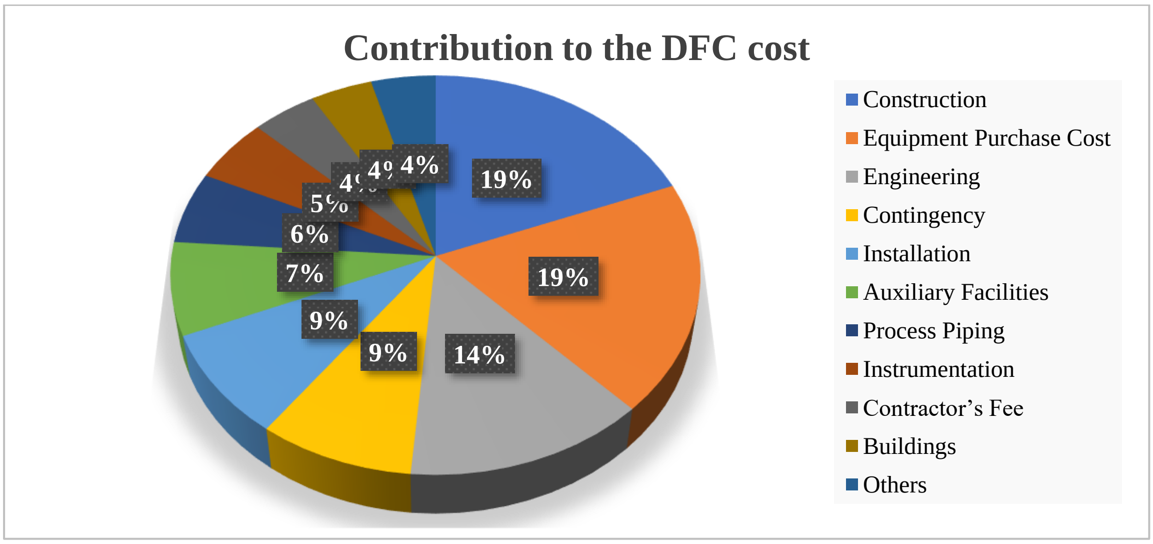

Figure S2. Contribution to the Direct Fixed Capital (DFC) cost

Supplementary Tables

Table S1. CAPEX calculation and results

Notation	Description	Formula	US$
A	Total Plant Direct Cost (TPDC)		
	• Equipment Purchase Cost (PC)	(see Supplementary Table S3)	9,655,000
	• Installation		4,404,000
	• Process Piping	0.31 × PC	2,993,000
	• Instrumentation	0.28 × PC	2,703,000
	• Insulation	0.03 × PC	290,000
	• Electrical	0.10 × PC	966,000
	• Buildings	0.22 × PC	2,124,000
	• Yard Improvement	0.10 × PC	966,000
	• Auxiliary Facilities	0.40 × PC	3,862,000
B	Total Plant Indirect Cost (TPIC)		
	• Engineering	0.25 × TPDC	6,991,000
	• Construction	0.35 × TPDC	9,787,000
C	Total Plant Cost (TPC)	TPDC + TPIC	44,740,000
D	Contractor's Fee & Contingency (CFC)		
	• Contractor's Fee	0.05 × TPC	2,237,000
	• Contingency	0.10 × TPC	4,474,000
E	**Direct Fixed Capital Cost (DFC)**	**TPC + CFC**	**51,451,000**
F	Working Capital (WC)<br>(Expenses to cover for 30 days of major OPEX costs)	Combined costs of labor, raw materials, utilities, and waste treatment, for 30 days	1,644,000
G	Start-up and Validation Cost	0.05 × DFC	2,573,000
H	Up front R & D	$1,000/section	5,000
I	**Total Capital Investment (CAPEX)**	E + F + G + H	**55,672,000**

Table S2. Contributions to the OPEX

Cost Item	Value (US$ y^{-1})	Note
Raw Materials	17,042,000	See Supplementary Table S4
Land Lease	7,075,000	Based on [67]
Depreciation	4,888,000	See Supplementary info. S1
Facility-Dependent	4,786,000	See Supplementary info. S2
Labor-Dependent	730,000	Simulated Result
Utilities	266,000	See Supplementary Table S6
Laboratory/QC/QA	109,000	15% of total labor cost
Waste Treatment/Disposal	32,000	See Supplementary Table S8
TOTAL	34,927,000	

Table S3. Equipment Details

Name	Type	Units	Size		Installation Factor	Unit Price ($/Unit)	Total Price ($)
DS-101	Disk-Stack Centrifuge	11	100	m^3/h	0.5	431,000	4,741,000
XD-101	Extruder	3	350	kg/h	0.5	206,000	618,000
V-102	Flat Bottom Tank	1	4,696	L	0.5	32,000	32,000
FAD-101	Flow Adjustor	3	726	MT/h	0.5	1,000	3,000
GR-101	Grinder	1	550	kg/h	0.5	77,000	77,000
MX-102	Mixer	4	544	MT/h	0.5	1,000	4,000
MX-101	Mixer	4	550	MT/h	0.5	1,000	4,000
RP-101	Raceway Pond	16	3,648	m^3	0.5	80,000	1,280,000
V-101	Receiver Tank	1	7,013	L	0.3	63,000	63,000
SDR-101	Spray Dryer	2	79,522	L	0.5	212,000	424,000
R-101	Agitator	1	4,635	L	0.3	134,000	134,000
WSH-101	Washer	43	2,456	kg/h	0.5	8,000	344,000

Table S4. Summary of raw material costs

Materials	Annual Amount	Unit Cost	Annual Cost ($)
Glycerol	273.6 MT	0.50 $/kg	136,800
Spirulina inoculum	182.4 MT	15.0 $/kg	2,736,000
Tap water	2,889 m^3	0.85 $/$m^3$	2,456
Zarrouk medium (Table S5)	1.77×10^8 L	0.08 $/L	14,166,300
TOTAL			17,041,555

Table S5. Composition of Zarrouk medium

Chemicals	Concentration	Unit
Calcium Chloride(aq)	0.04	g/L
Dipotassium Hydrogen Phosphate	0.5	g/L
EDTA Disodium	0.08	g/L
Iron(II) Sulfate(aq)	0.01	g/L
Magnesium Sulfate(aq)	0.2	g/L
Micronutrients (Each in g/L: H_3BO_3, 2.86; $MnCl_2.4H_2O$, 1.81; $ZnSO_4.4H_2O$, 0.222; $CuSO_4.5H_2O$, 0.079; Na_2MoO_4, 0.0177)	1	ml
Potassium Sulfate	1	g/L

Chemicals	Concentration	Unit
Sodium Bicarbonate	16.8	g/L
Sodium Chloride	1	g/L
Sodium Nitrate	2.5	g/L
Tap water	1	L

Table S6. Summary of the prices of utilities

Utility	Annual Amount	Unit Cost	Annual Cost ($)
Electricity (standard power)	2,489,495 kWh	0.1 $/kWh	248,949
Cooling water (25-30 °C)	21,749 MT	0.1 $/MT	2,175
Chilled water (5-10 °C)	16,555 MT	0.5 $/MT	8,277
Steam (~152 °C)	206 MT	32 $/MT	6,604
TOTAL			266,006

Table S7. Power demand breakdown by section

Section	Equipment	Demand (kWh/y)
Harvesting	Centrifuge machine	1,600,495.17
Cultivation	Raceway Pond	479,039.69
Treatment	Grinding machine	250,910.07
Plastic Making	Agitator and Extrusion machines	159,049.90
TOTAL		2,489,494.83

Table S8. Summary of waste treatment/disposal cost

Waste Category	Annual Amount	Unit Cost	Annual Cost ($)
Aqueous Liquid			
• Adj. water out	159,226 m^3	0.11 $/$m^3$	17,515
• P-9:CIP-1(Cleaning Step #1)	2,870 MT	5.00 $/MT	14,348
TOTAL			31,863

Other Supplementary Information

S1: Depreciation

Depreciation is calculated as part of the OPEX. Depreciation is an income tax deduction that represents a fixed capital loss which is mostly due to equipment wear out and obsolescence. It may be considered as a time-dependent operating cost, spread over a predefined depreciation period. For each section, SuperPro Designer depreciates the fraction of DFC that is assigned to this project and has not been depreciated already minus its salvage value at the end of the depreciation period. The startup and validation cost can also be depreciated if specified. In the general case, the total depreciable amount, d_{tot}, of a section's assets over the entire depreciation period is calculated as:

$$d_{tot} = \sum_{1}^{N} d_k = B - S$$

Where:

$B = f_p \times UDFC + C_s$

$S = f_s \times f_p \times UDFC$

and:

- d_k is the depreciable amount of a section's assets in year k,
- N is the depreciation (recovery) period,
- B is the cost basis of a section's assets (the cost right before the project starts),
- S is the salvage value of the section's assets at the end of the depreciation period (i.e., S is the re-selling price of plant equipment after the usual project lifespan) = 5% of DFC,
- f_p is the fraction of a section's DFC (direct fixed capital) that is assigned to this project,
- UDFC is the undepreciated DFC of a section (i.e., the fraction of a section's DFC that has not been depreciated already),
- C_s is the startup & validation cost of a section, and
- f_s is the salvage fraction of the entire DFC.

S2: Facility-Dependent Cost (FDC)

FDC is calculated as part of the OPEX. This accounts for additional costs related to the use of a facility. In cases of new (green-field) designs, where no prior experience on the use of equipment exists, this is typically calculated as the sum of the costs associated with equipment maintenance, depreciation of the fixed capital cost, and miscellaneous costs such as insurance, local (property) taxes and possibly other overhead-type of factory expenses.

FDC = Maintenance + Depreciation + Miscellaneous

Where:

- Maintenance was estimated based on the specified maintenance factors and calculated time utilization fractions for all equipment,
- Depreciation as specified in section S1, and
- Miscellaneous = Insurance (1% of DFC) + Local Taxes (2% of DFC) + Factory Expense (5% of DFC).
